# Acupuncture for low back pain due to spondylolisthesis: study protocol for a randomized controlled pilot trial

**DOI:** 10.1186/1745-6215-15-105

**Published:** 2014-04-02

**Authors:** Hyun-jong Lee, Jung-Chul Seo, Min-Ah Kwak, Sung-Hoon Park, Bo-Mi Min, Min-su Cho, ImHee Shin, Jin-yong Jung, Woon-seok Roh

**Affiliations:** 1 Department of Acupuncture & Moxibustion, College of Oriental Medicine, Daegu Haany University, Daegu, 165 Sang-dong, Suseong-gu, Daegu 706-060, Republic of Korea; 2 Comprehensive and Integrative Medicine Institute, Daegu, 3056-6 Daemyeong 4-dong, Nam-gu, Daegu 705-718, Republic of Korea; 3 Department of Internal Medicine, College of Oriental Medicine, Daegu Haany University, Daegu, 165 Sang-dong, Suseong-gu, Daegu 706-060, Republic of Korea; 4 Department of Anesthesiology and Pain Medicine, School of Medicine, Catholic University of Daegu, Daegu, 3056-6 Daemyeong 4-dong, Nam-gu, Daegu 705-718, Republic of Korea; 5 Department of Medical Statistics, School of Medicine, Catholic University of Daegu, Daegu, 3056-6 Daemyeong 4-dong, Nam-gu, Daegu 705-718, Republic of Korea

**Keywords:** Acupuncture, Interlaminar epidural steroid injection, Spondylolisthesis

## Abstract

**Background:**

Spondylolisthesis is the major cause of refractory low back pain. There are many studies of the surgical treatment of spondylolisthesis, but few of conservative treatments. There is also no optimal conservative treatment protocol, however, low back pain caused by low-grade spondylolisthesis is controlled with non-surgical pain management. Acupuncture has become a useful method for treating low back pain, but there has not been any study of its efficacy in relation to spondylolisthesis. This study was designed to establish the feasibility of a randomized controlled trial and the safety of acupuncture for low back pain due to low-grade spondylolisthesis.

**Methods/Design:**

The study is a randomized controlled pilot clinical trial of five weeks duration. Fourteen patients will be recruited and randomly allocated to two groups: an acupuncture plus interlaminar epidural steroid injection group (experimental group), and an interlaminar epidural steroid injection group (control group). All patients will be administered an interlaminar epidural steroid injection once a week for three weeks (three injections in total), but only the experimental group will receive additional treatment with three acupuncture sessions a week for three weeks (nine acupuncture sessions in total). The primary outcome will be measured by the visual analogue scale (VAS). Our primary end point is three-week VAS. The secondary outcome will be measured using the PainVision system, the short-form McGill Pain Questionnaire, and the Oswestry Disability Index. Assessments will be made at baseline and at one, three and five weeks thereafter (that is, the five-week assessment will be made two weeks after treatment cessation).

**Discussion:**

This randomized controlled pilot trial will inform the design of a further full-scale trial. The outcomes will provide some resources for incorporating acupuncture into existing pain management methods such as interlaminar epidural steroid injection in low-grade spondylolisthesis.

**Trial registration:**

This trial is registered with the US National Institutes of Health Clinical Trials registry: NCT01909284.

## Background

Spondylolisthesis is defined as anterior migration, or slippage, of a vertebral body relative to the next caudad vertebral segment [1]. Spondylosis and spondylolisthesis occur most commonly in the fifth lumbar vertebral body, but may occur at more cephalad lumbar levels. Patients with L4 spondylosis and spondylolisthesis are more frequently symptomatic [2,3]. The symptoms clinically associated with spondylolisthesis are low back pain and pain, numbness, or weakness in the legs or lower extremities [4]. The classification systems of Wiltse-Newman [1] and Marchetti and Bartolozzi [5] are the most widely used. In terms of describing the actual severity of spondylolisthesis, the Meyerding scale is the most widely recognized method. The magnitude of spondylolisthesis is measured by dividing the slip distance by the caudad body width and expressed as a percentage. The slip percentage is then categorized according to the Meyerding scale as follows: Grade 0, no slip; Grade I, 1 to 25%; Grade II, 26 to 50%; Grade III, 51 to 75%; Grade IV, 76 to 100%; Grade V: complete slippage. Both the slip percentage and Meyerding grade are reliable measures of severity [6,7].

Many patients with low-grade spondylolisthesis (Meyerding grade 0, I, II) can be treated conservatively [8]. Most patients with spondylolisthesis do not worsen with time and rapid deterioration is very rare [9]. Physical therapy and pain management are the mainstays of conservative treatment, and should be the initial course of action in most cases of spondylolisthesis, with or without neurological symptoms [8]. Most physicians begin with antiinflammatory medications and physical therapy, including aerobic conditioning, provided there are no gastrointestinal contraindications. If symptoms persist beyond four to six weeks, patients may benefit in the short term from a course of epidural steroid injections. Benefits may include reduced lower back and radiculopathy-associated pain [4,10].

Acupuncture has become a popular alternative treatment modality for patients with low back pain, which is the major symptom of spondylolisthesis. Indeed, acupuncture has been shown to be effective in relieving low back pain [11,12]. Leibing *et al.*[13], Haake *et al.*[14], and Brinkhaus *et al.*[15] all reported a significant difference in their primary outcome measures–Visual Analog scale (VAS), Roland Morris Disability Questionnaire, and McGill Pain Index. Witt *et al.*[16] also reported a significant improvement in pain, function, and quality of life after acupuncture treatment when compared to routine care, at three months. However, no published trials have evaluated the efficacy and safety of acupuncture in patients with low back pain due to spondylolisthesis.

In the evaluation of the clinical and radiographic result of the transforaminal lumbar interbody fusion in degenerative and isthmic lower-grade spondylolisthesis, there were 7.6% serious postoperative complications observed, which required operative revision [17]. Acupuncture would be helpful in patients with spondylolisthesis as a safe conservative treatment without severe adverse events as can happen in some surgical procedures, such as transforaminal lumbar interbody fusion. The primary aim of this trial is to establish the feasibility of a randomized controlled trial and the safety of acupuncture for reducing low back pain due to low-grade spondylolisthesis and improving function compared with usual interlaminar epidural steroid injection.

### Hypotheses

In this randomized controlled two-arm clinical trial, we will evaluate the effect of acupuncture as an adjunct therapy to spondylolisthesis. The primary hypothesis is that acupuncture treatment in addition to interlaminar epidural steroid injection reduces pain (as measured by VAS) significantly more than interlaminar epidural steroid injection only. The secondary hypotheses are that: the score of the PainVision system (PS-2100, Nipro Corporation, Osaka, Japan), is significantly lower in the experimental group than that in the control group; additional acupuncture treatment reduces the score on the short-form McGill Pain Questionnaire (SF-MPQ) significantly more than interlaminar epidural steroid injection alone; interlaminar epidural steroid injection combined with acupuncture is significantly better than interlaminar epidural steroid injection alone in reducing the score on the Oswestry Disability Index (ODI).

## Methods/Design

### Design

The study is a randomized controlled pilot clinical trial. It is designed to assess the efficacy and safety of acupuncture treatment in patients with low back pain due to low-grade spondylolisthesis. The protocols used adhere to the principles of the Declaration of Helsinki and have been approved by the institutional review board of Daegu Catholic University Hospital (IORG0004453), where the study will take place. The trial is registered with the US National Institutes of Health Clinical Trials registry. Written consent will be obtained from each participant before any treatment is given.

**Figure 1:**
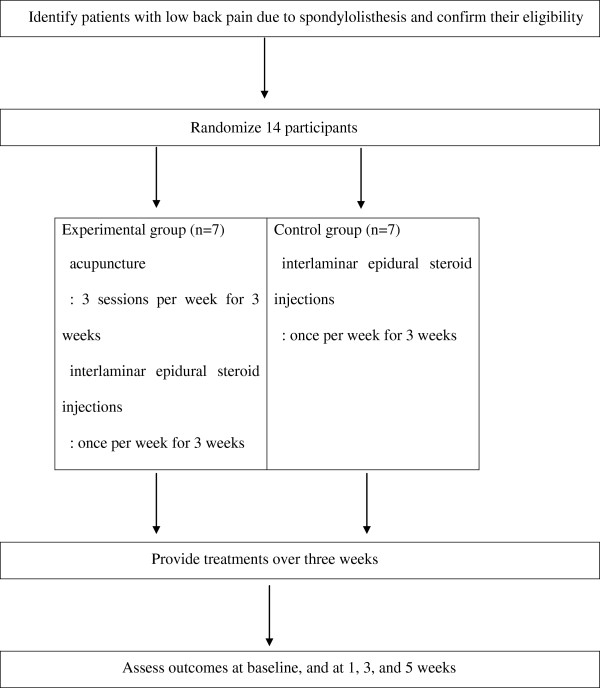
A flow chart of the trial process.

The outcome assessment and statistical analyses will be performed by professionals blinded to the assignment of patients. The trial process is presented in Figure 1. The trial will run for five weeks. Patients will be allocated to two groups; the control group will only receive three interlaminar epidural steroid injections and the experimental group will receive all three steroid injections as well as nine acupuncture sessions (three sessions a week forthree weeks). Assessments will be made at baseline, and again at one, three and five weeks thereafter. The week five assessment will be performed two weeks after treatment cessation.

### Recruitment

Participants will be recruited through advertisements on hospital websites and bulletin boards. If patients are interested in participation, they will be invited to visit the hospital for a screening meeting. Their eligibility will be determined by an anesthesiologist through physical and radiological examinations. If eligible, they will be guided through the informed consent process. After written consent is obtained, a study researcher will randomly allocate the participants to one of the two groups. Treatments will be scheduled after randomization.

### Participants

One of the main objectives of this study is to provide an estimate of the sample size required for the full-scale randomized controlled clinical trial. We plan to recruit 14 patients into this pilot study. A target of 14 patients with spondylolisthesis has been set.

The inclusion criteria are that participants must: be aged 18 to 65 years, have Meyerding Grade I to II spondylolisthesis, have low back pain of at least one-year duration, be available for possible follow-up during the clinical trial, and provide written informed consent voluntarily.

The exclusion criteria are: cauda equina syndrome, persistently exacerbated symptoms, progressive neurologic signs (sensory or motor changes); previous spine surgery; senile dementia, impaired cognitive function or other cerebral disease, severe psychiatric or psychological disorders; severe, concomitant disease (neuromuscular scoliosis, neurodegenerative disease); all contraindications to corticosteroid injection (e.g., insulin-dependent diabetes); alcohol/drug abuse; significant renal or hepatic disease; pregnant, lactating or planning a pregnancy; hypersensitive reaction to acupuncture treatment; inability to comprehend or express oneself in the Korean language; an individual deemed to be ineligible by a physician; or refusal to participate in the trial or to provide informed consent.

### Randomization

Patients will be randomized using a computerized random number generator by an independent statistician blinded to patient assignment. Block randomization will be performed once a participant is confirmed to be eligible and their written informed consent has been obtained.

### Interventions

Patients will be randomly divided into two treatment groups: an experimental group (acupuncture plus interlaminar epidural steroid injection group) and a control group (interlaminar epidural steroid injection group). The interlaminar epidural steroid injections will be administered once per week for three weeks (three times in total) and the acupuncture sessions performed three times per week for three weeks (nine times in total).

### Interlaminar epidural steroid injection

The lumbar interlaminar epidural injections will be administered by an anesthesiologist under fluoroscopy in a sterile procedure room in an outpatient pain management department. The lumbar interlaminar epidural space will be identified by the loss of resistance technique and by fluoroscopic visualization with confirmation with a non-ionic contrast medium. The epidural space will be entered at the level of spondylolisthesis or one space above/below the level of spondylolisthesis. After confirmation of needle placement, 10 mL mepivacaine hydrochloride 0.1% (preservative free) and 5 mg non-particulate dexamethasone will be administered by injection.

### Acupuncture treatment

The following acupoints will be used: GV3 (unilateral), BL23, BL24, BL25, GB30, GB31, BL40, and BL60 (bilateral). In total, 15 acupoints will be used. Sterilized disposable acupuncture needles (DongBang Acupuncture Inc., Sungnam, Korea) 0.25 mm × 40 mm in size will be manually inserted into each of the 15 acupoints. After needle insertion, the Deqi sensation will be induced by manual stimulation, and four acupoints (bilateral BL23, BL25) will be stimulated by an electro-acupuncture device (ES-160, Ito Co. Ltd., Tokyo, Japan). The needles will be inserted for 20 ± 5 minutes and then removed.

### Data collection

In this study, the primary outcome will be measured by VAS. The secondary outcome will be measured by the PainVision system, the SF-MPQ and ODI. Both the primary and secondary outcomes will be measured at baseline, and at one, three and five weeks thereafter. The schedule of treatment and outcome assessments is presented in Table 1.

**Table 1:** Schedule of treatments and outcome measures throughout the trial

		**Baseline**	**Treatment period**									**Follow-up period**	
		**0 week**	**1 week**			**2 week**			**3 week**			**4 week**	**5 week**
Measures	VAS	√	√						√				√
	Pain Vision	√	√						√				√
	SF-MPQ	√	√						√				√
	ODI	√	√						√				√
	Safety	√							√				
**Treatments**	Interlaminar epidural steroid injection		√			√			√				
	acupuncture		√	√	√	√	√	√	√	√	√		

*ODI*, Oswestry Disability Index; *SF-MPQ*, short-form McGill Pain Questionnaire; *VAS*, visual analogue scale.

### Primary outcome measurement

#### VAS

Pain intensity will be assessed using a10 cm VAS of subjective pain assessment. Each patient will rate their pain on a scale of 0 to 10 (0 being an absence of pain and 10 being the worst pain imaginable) [18,19]. VAS measurements will be made at baseline, and at one, three and five weeks. Our primary end point is three-week VAS.

### Secondary outcome measurements

#### PainVision

PainVision is a system used for the quantitative analysis of perception and pain; it has recently been introduced in the field of pain and anesthesiology [20,21]. The PainVision system consists of four devices: (1) the main PainVision system unit, (2) a personal computer connected to the PainVision system, (3) sensors, and (4) a printer. The specific protocols for using the systems are as follows [22]: first, sensors that transmit an electric current will be attached to the right medial forearm. The current perception threshold that indicates the pain threshold of each subject will be measured three times, and the mean values will be used for analysis; Second, the pain compatible electrical current will be measured by a gradually increasing pulsed current applied to the right medial forearm. The pain due to spondylolisthesis and the magnitude of electric stimulation are believed equal. The pain-compatible electrical current will be measured three times, and the mean values will be used for analysis. On the basis of these measurements, pain intensity will be calculated using the following equation:

$$\begin{array}{ll} \;\text{Pain intensity}=100 & \times \left(\text{pain}‒\text{compatible eletrical current}\right)\\ & \;\left(‒\text{current perception threshold}\right)\\ & \;/\text{current perception threshold} \end{array}$$

### SF-MPQ

The SF-MPQ, a shorter version of the MPQ, is a multidimensional measure of perceived pain in adults with chronic pain [23,24]. The main component of the SF-MPQ consists of 15 descriptors (11 sensory, 4 affective) from the original MPQ. The descriptors are rated on an intensity scale as 0 = none, 1 = mild, 2 = moderate, or 3 = severe. The SF-MPQ also includes the Present Pain Intensity (PPI) scale. The PPI scale is a measure of the magnitude of pain experienced by an individual and a numeric-verbal combination that indicates overall pain intensity and includes 6 levels: 0 = none, 1 = mild, 2 = discomforting, 3 = distressing, 4 = horrible, and 5 = excruciating [25]. Higher numbers indicate more severe pain. The SF-MPQ will be measured at baseline and at one, three and five weeks.

### ODI

The ODI is one of the most frequently used tools for measuring disability related to low back pain [26]. The ODI contains 10 questions about daily activities, including inventories of pain intensity, personal care, lifting, walking, sitting, standing, sleeping, sexual life, social life, and traveling. Each question is rated on a scale from 0 to 5 points. The ODI scores range from 0 to 50. Higher scores indicate greater disability. The validated Korean version of the ODI [27] will be measured at baseline, and at one, three and five weeks.

### Safety

We will confirm the safety of acupuncture by determining the red blood cell (RBC) count, hemoglobin level, platelet count, mean corpuscular volume (MCV), mean corpuscular hemoglobin (MCH), mean corpuscular hemoglobin concentration (MCHC), hematocrit (Hct), total white blood cell (WBC) count, erythrocyte sedimentation rate (ESR), aspartate aminotransferase (AST), alanine aminotransferase (ALT), serum urea nitrogen (BUN), creatinine level, serum sodium level, serum potassium level, and serum chloride level. All patients were evaluated two times, including the screening visit and after the termination of acupuncture.

Any reported adverse events will be recorded throughout the study and vital signs will be monitored at each visit. The patients will be requested to voluntarily report information about adverse events, and the researcher will confirm the occurrence of adverse events through methods such as a medical interview. Details about adverse events, such as the date of occurrence, degree of adverse events, causal relationship with the treatment, other treatments or medications that are suspected to cause the adverse event, and treatment of the adverse event, will be recorded in detail.

### Withdrawal and dropout

All patients will have the right to withdraw from the study at any time. Participation will be ended at any stage if the patient refuses to continue, withdraws their consent, or violates the inclusion or exclusion criteria or the trial protocol. The trial will be stopped if the principal investigator believes that there are unacceptable risks of serious adverse events.

### Statistical analysis

The statistical significance level will be set at 5%, and the data will be analyzed using intention-to-treat and per protocol approaches. Data will be processed with the last observation carried forward method for the intention-to-treat analysis, and will be performed using IBM SPSS Win ver. 19.0 statistical software (IBM Co., Armonk, NY, USA).

All demographic and clinical characteristics of the subjects (such as sex, age, and weight) will be processed based on descriptive analyses. Quantitative data will be presented as average, standard deviation, median value, and range. Qualitative data will be presented as the frequency and percentage. The study will identify the comparative equivalence of demographic variables and clinical characteristics between the experimental and control groups.

In order to identify differences in VAS, PainVision scores, SF-MPQ and ODI scores between the experimental and control group based on time (baseline, weeks one, three and five), a repeated-measure twofactor analysis will be performed to identify differences between groups, differences within each group based on time, and the effects of the interaction of the variables based on group. If the interaction between group and time is statistically significant, the point at which the pattern of results between the two groups changes will be checked.

The Chi-square test will be used to compare groups and the incidence frequency of adverse events related to acupuncture and interlaminar epidural steroid injection.

## Discussion

Recently, there has been increased interest in the use of acupuncture for treating diseases [28]. Many studies have reported that acupuncture is suitable for providing pain relief and improving function in patients with low back pain [29]. However, there is currently no evidence supporting the use of acupuncture in patients with low back pain due to low-grade spondylolisthesis. Our pilot study will evaluate the feasibility of further research on acupuncture as an effective and safe treatment for reducing pain and improving function in these patients. Although many surgical options exist for the treatment of spondylolisthesis, it is generally agreed that in most cases, conservative treatment should be attempted before surgical intervention.

Many clinical trials have compared surgical treatment methods. However, no randomized clinical trial has established an optimal non-operative treatment protocol [4]. Our study is related to low-grade spondylolisthesis to apply conservative treatment at first. This randomized controlled pilot trial will inform the design of a further full-scale trial. The outcomes will provide some resources for incorporating acupuncture into existing pain management programs such as interlaminar epidural steroid injection for treating low-grade spondylolisthesis.

### Trial status

This trial is currently recruiting participants. Enrollment and trial completion is expected to be finished by the end of 2013.

## Abbreviations

ALT: alanine aminotransferase; AST: aspartate aminotransferase; BUN: serum urea nitrogen; ESR: erythrocyte sedimentation rate; Hct: hematocrit; MCH: mean corpuscular hemoglobin; MCHC: mean corpuscular hemoglobin concentration; MCV: mean corpuscular volume; ODI: Oswestry Disability Index; PPI: Present Pain Intensity; RBC: red blood cell; SF-MPQ: Short form McGill pain questionnaire; VAS: Visual analogue scale; WBC: white blood cell.

## Competing interests

The authors declare that they have no competing interests.

## Authors’ contributions

HJL: conception, design of the study and manuscript writing, JCS: conception, design of the study and manuscript writing. MAK: conception of the study. SHP: conception of the study. BMM: design for the protocol. MSC: design for the protocol. IHS: critical revision of the manuscript. JYJ: critical revision of the manuscript. WAR: conception, design, and critical revision of the manuscript. All authors read and approved the final manuscript.
